# Impact of HLA Epitope Matching on Outcomes After Unrelated Bone Marrow Transplantation

**DOI:** 10.3389/fimmu.2022.811733

**Published:** 2022-03-03

**Authors:** Makoto Iwasaki, Junya Kanda, Hidenori Tanaka, Takero Shindo, Takahiko Sato, Noriko Doki, Takahiro Fukuda, Yukiyasu Ozawa, Tetsuya Eto, Naoyuki Uchida, Yuta Katayama, Keisuke Kataoka, Takahide Ara, Shuichi Ota, Makoto Onizuka, Yoshinobu Kanda, Tatsuo Ichinohe, Yoshiko Atsuta, Satoko Morishima

**Affiliations:** ^1^Department of Hematology and Oncology, Graduate School of Medicine, Kyoto University, Kyoto, Japan; ^2^HLA Foundation Laboratory, Kyoto, Japan; ^3^Department of Hematology and Oncology, Nagoya University Graduate School of Medicine, Nagoya, Japan; ^4^Hematology Division, Tokyo Metropolitan Cancer and Infectious Diseases Center, Komagome Hospital, Tokyo, Japan; ^5^Department of Hematopoietic Stem Cell Transplantation, National Cancer Center Hospital, Tokyo, Japan; ^6^Department of Hematology, Japanese Red Cross Aichi Medical Center Nagoya Daiichi Hospital, Nagoya, Japan; ^7^Department of Hematology, Hamanomachi Hospital, Fukuoka, Japan; ^8^Department of Hematology, Federation of National Public Service Personnel Mutual Aid Associations, Toranomon Hospital, Tokyo, Japan; ^9^Department of Hematology, Hiroshima Red Cross Hospital and Atomic-bomb Survivors Hospital, Hiroshima, Japan; ^10^Division of Hematology, Department of Medicine, Keio University School of Medicine, Tokyo, Japan; ^11^Department of Hematology, Hokkaido University Hospital, Sapporo, Japan; ^12^Department of Hematology, Sapporo Hokuyu Hospital, Sapporo, Japan; ^13^Department of Hematology and Oncology, Tokai University School of Medicine, Isehara, Japan; ^14^Division of Hematology, Jichi Medical University, Tochigi, Japan; ^15^Department of Hematology and Oncology, Research Institute for Radiation Biology and Medicine, Hiroshima University, Hiroshima, Japan; ^16^Japanese Data Center for Hematopoietic Cell Transplantation, Nagoya, Japan; ^17^Department of Registry Science for Transplant and Cellular Therapy, Aichi Medical University School of Medicine, Nagakute, Japan; ^18^Division of Endocrinology, Diabetes and Metabolism, Hematology, Rheumatology (Second Department of Internal Medicine), Graduate School of Medicine, University of the Ryukyus, Nishihara, Japan

**Keywords:** unrelated bone marrow transplantation, epitope, HLAMatchmaker, acute GVHD, high-risk mismatch

## Abstract

The significance of antibody-identified epitopes stimulating humoral alloimmunity is not well understood in the identification of non-permissive human leukocyte antigen (HLA) mismatching patterns in hematopoietic stem cell transplantation (HSCT). This was a retrospective study in a cohort of 9,991 patients who underwent their first HSCT for hematologic malignancies from unrelated bone marrow donors in the Transplant Registry Unified Management Program (TRUMP). HLA eplet mismatches (EMM) were quantified using HLAMatchmaker (HLAMM). The median age of patients was 48 years (range, 16 to 77). The number of EMM in recipient-donor pairs in our study population ranged from 0 to 37 in HLA class I (median, 0) and 0 to 60 in HLA class II (median, 1). In addition to the known high-risk mismatch patterns in the Japanese cohort, HLA-C EMM in the GVH direction was associated with a significantly higher risk for grade III-IV aGVHD, leading to a higher risk of non-relapse mortality and lower overall survival (compared with HLA-C matched patients, HR 1.67, 95% CI 1.44–1.95; HR 1.39, 95% CI 1.25–1.54; HR 1.20, 95% CI 1.10–1.30, respectively). HLAMM-based epitope matching might be useful for identifying patients who are at high risk for serious complications after HSCT from HLA mismatched unrelated donors.

## Introduction

Human leukocyte antigen (HLA) disparity causes an immune reaction between recipient and donor cells after hematopoietic stem cell transplantation (HSCT). Extensive studies have demonstrated that HLA disparity is associated with a higher risk of graft-versus-host disease (GVHD) and a longer time to engraftment, leading to a poor prognosis of HLA-mismatched recipient-donor pairs in transplantation from unrelated donors. Early studies focused on the number of mismatched HLA antigens or alleles, and the quantification of HLA antigens and alleles is prioritized in donor selection (1, 2). HLA locus matching and the mismatched direction are also potent prognostic factors and are taken into consideration in clinical settings (3–6). Prognostic HLA mismatching patterns have also been investigated based on HLA supertypes and haplotypes (7–9). However, it is not yet fully understood what underlies the heterogeneous effect of HLA mismatching on HSCT outcomes.

To clarify the heterogeneity in the immunogenicity of HLA disparity, there have been many attempts to identify specific patterns of amino acid substitution associated with a poor prognosis after HSCT (10–17). A previous study from the Japanese Marrow Donor Program (JMDP) reported non-permissive HLA allele mismatch combinations based on the association with grade III-IV severe acute GVHD (aGVHD) which is a solid marker for alloreactivity in HSCT (13). A subsequent study from JMDP showed a significant association between patient mismatched HLA-C*14:02 and severe aGVHD (17). To understand the immunogenicity of amino acid sequences in mismatched HLA pairs, several methods have been developed to predict epitopes recognized by the immune system (18–21). Duquesnoy et al. established the HLAMatchmaker (HLAMM) algorithm based on in silico prediction combined with an *in vitro* antigen-antibody reaction to identify B cell epitopes presented as triplets, which they called ‘eplets’ (22, 23). Duquesnoy et al. could not find significant association with eplet mismatching with transplantation outcomes in unrelated bone marrow transplantation (UR-BMT) (24). HLA-haploidentical HSCT (haplo-HSCT) using high-dose posttransplant cyclophosphamide-based GVHD prophylaxis (PTCy) has been widely used because PTCy suppresses alloreactive T cells and prevents acute and chronic GVHD (25–28). Several studies investigated the association of eplet matching in haplo-HSCT using PTCy. Rimando et al. reported that eplet matching for HLA class II in the GVH direction was associated with the incidence of relapse for patients received haplo-HSCT using PTCy (29). Zou et al. demonstrated that HLA-B eplet mismatching was associated with aGVHD for patients received haplo-HSCT using PTCy in single center study, but they could not validate the results in registry data of Center for International Blood and Marrow Transplantation Research (CIBMTR) (30, 31), The impact of eplet mismatching in hematopoietic stem cell transplantation is still being actively discussed.

We aimed to understand the significance of eplet matching in identifying non-permissive mismatching patterns in HLA allele-mismatched patient-donor pairs. We investigated the association of HLAMM-based eplet mismatches (EMM) with outcome after UR-BMT using Japanese registry data.

## Materials and Methods

### Population

All transplantation data in Japan are annually collected at the Japanese Data Center for Hematopoietic Cell Transplantation (JDCHCT). The study was conducted according to the Declaration of Helsinki and was approved by the institutional review boards at Kyoto University Hospital, where this study was organized, and the Data Management Committees of the Japanese Society for Transplantation and Cellular Therapy (JSTCT) and JDCHCT. All patients provided their written informed consent for research.

### Inclusion and Exclusion Criteria

From the registry database of TRUMP, patients aged 16 years or older who underwent their first allogeneic stem cell transplant using bone marrow graft from unrelated donors for hematologic malignancies between 2000 and 2018 were included. Patients for whom data on recipient and donor HLA-A, -B, -C, and -DRB1 alleles, date of last follow-up or patient outcomes were lacking were excluded. We also checked the existence of HLA-A, -B, -C, and -DRB1 alleles in an HLA allele frequency database provided by the Japanese Society for Histocompatibility and Immunogenetics (http://jshi.umin.ac.jp/standarization/index.html), and excluded recipient-donor pairs which possessed non-existing HLA alleles in this database to exclude incorrect HLA information made by simple mistakes in registration.

### Prediction of DQB1 Aleles Based on a Maximum Probability Algorithm

High-resolution HLA-typing data were collected for the following HLA loci: A, B, C, DRB1, and DQB1. For recipient-donor pairs that lacked information about HLA-DQB1 alleles, we used a two-step method for estimating DQB1 allele (**Supplementary Figure S1**). First, haplotypes of HLA-A, -B, -C, and -DRB1 loci were estimated using a maximum probability algorithm (MPA) as described previously (9). Briefly, eight possible haplotype combinations were determined based on the results of HLA-A, -B, -C, and -DRB1 genotyping in each patient. The probabilities of the 8 haplotype combinations were calculated using haplotype frequency data from a family study in a Japanese population (HLA laboratory; http://hla.or.jp/med/frequency_search/ja/haplo/). The haplotype combination with the highest probability among the 8 combinations was used as the predicted haplotype of the patient. Next, the probability of the HLA-DQB1 allele for each determined haplotype was also estimated using haplotype frequency data of HLA-B, -DRB1, and -DQB1. Only haplotypes and each DQB1 allele that were determined to have a likelihood ratio of 80% for both donors and recipients were included.

### Quantification of Epitope Mismatch

For HLA mismatched donor-recipient pairs, EMM was quantified using HLAMatchmaker software (HLA-Matchmaker ABC Eplet Matching version 3.1 and DRDQDP Eplet Matching version 3.1; http://www.hlamatchmaker.net) and a Python script (available at https://github.com/cliu32/hla-mm) adjusted for HLAMatchmaker version 3.1 in the GVH and HVG directions separately. Because the HLAMM database lacked information on the HLA-C*01:55 allele, we investigated amino acid substitutions and epitope matching based on the IMGT database (IMGT/HLA Allele Query Form; https://www.ebi.ac.uk/ipd/imgt/hla/allele.html) for two patients with HLA-C*01:55 allele. These calculations were performed with Python 3.7.9.

### HLA Eplet-Based Subgroup

To understand the impact of HLA eplet mismatching in class I and class II on HSCT outcomes, we divided recipient-donor pairs with HLA allele mismatches (AMM) into 5 groups: serotype-matched recipient-donor pairs with low EMM in class I and class II (SM CI/II-lo), serotype-mismatched recipient-donor pairs with low EMM in class I and class II (SMM CI/II-lo), recipient-donor pairs with high class I EMM and low class II EMM (CI-hi/CII-lo), recipient-donor pairs with low class I EMM and high class II EMM (CI-lo/CII-hi), and recipient-donor pairs with high EMM in class I and class II (CI/II-hi).

To understand the effect of HLA-C locus matching on HSCT outcomes, we further divided patients into five groups based on HLA-C matching for alleles, antigens, eplets and high-risk substitutions (AMM S/EM: HLA-C allele-mismatched patients without antigen mismatches, and EMM, SMM EM: HLA-C antigen-mismatched patients without EMM, EMM HRM: HLA-C eplet-mismatched patients without high-risk mismatches, other HRMM; patients with high-risk mismatches other than patient mismatched HLA-C*14:02 in the GVH direction, rec1402MM; C*15:02 to C*14:02 and other recipient C*14:02 mismatch). High-risk mismatches other than patient mismatched HLA-C*14:02 included C*03:03 to C*15:02, C*03:04 to C*08:01, C*04:01 to C*03:03, C*08:01 to C*03:03, C*14:02 to C*03:04, and C*15:02 to C*03:04 (13, 17). KIR ligand mismatch in the GVH direction was defined as the donor’s KIR ligand for HLA-C not being shared by the patient’s ligand (32).

### Endpoint and Definitions

The primary outcome was the incidence of grade III-IV aGVHD. Secondary outcomes were overall survival (OS), relapse-free survival (RFS), relapse, grade II-IV aGVHD, non-relapse mortality (NRM), chronic GVHD (cGVHD), extensive cGVHD, time to neutrophil engraftment, and time to platelet engraftment. Relapse was defined based on morphological and clinical evidence of disease activity, and NRM was defined as the time to death without relapse. Acute and chronic GVHD were diagnosed and graded using standard criteria (33, 34). Time to neutrophil engraftment was defined as the first of 3 consecutive days with an absolute neutrophil count of 500 cells per microliter. Time to platelet engraftment was defined as the first of 2 weeks with a platelet count of 20,000 cells per microliter with no transfusion support in the past 2 weeks. The intensity of the conditioning regimen was classified as myeloablative if either total body irradiation >8 Gy, oral busulfan ≥9 mg/kg, intravenous busulfan ≥7.2 mg/kg, melphalan >140 mg/m2, or thiotepa ≥10 mg/kg was used in the conditioning regimen, and was otherwise classified as reduced intensity (35). Disease stage was defined as previously described (9).

### Statistical Considerations

Descriptive statistics were used to summarize the patient characteristics. Correlations among HLA allele disparities, HLA haplotype mismatching, and HLA epitope mismatching were assessed using Pearson’s correlation matrix. We conducted a multivariate analysis using a Cox proportional hazard regression for OS and the Fine and Gray competing risks regression model for relapse, NRM, grade II-IV aGVHD, grade III-IV aGVHD, cGVHD, extensive cGVHD, time to neutrophil engraftment, and time to platelet engraftment. We adopted the Fine-Gray model as the underlying regression model and computed the direct adjusted cumulative incidence curves for NRM and GVHD to account for competing risks (36). Competing events were death without relapse for relapse, relapse for NRM, death without engraftment for neutrophil or platelet engraftment, and death without GVHD for acute and chronic GVHD. For multiple comparisons, we applied P values of <0.005 as statistically significant. We applied this specific threshold based on two reasons: 1) This study intended to find non-permissive amino-acid substitutions other than those detected in the previous study from JMDP, so the same threshold for P value was applied in this study (13). 2) Several studies suggested that to lower the threshold from widely used P <0.05 to P <0.005 would be reasonable to reduce false positive rate and to maintain reproducibility of the study (37, 38). We carried out a multivariate analysis for the GVH and HVG directions separately because HLA allelic mismatches at each locus are highly correlated between the GVH and HVG directions. The covariates listed in **Table 1** were included in the final multivariate model regardless of their statistical significance in any univariate models. Because PTCy was not approved as GVHD prophylaxis for UR-BMT in Japan at the time of last follow-up date of this study, we did not consider PTCy usage as covariates in the multivariate analysis in spite of 8 patients (0.08%) registered as using PTCy. All statistical analyses were performed with Stata version 15.1 software (Stata Corp., College Station, TX) and R version 4.0.2.

**Table 1:** Patient characteristics.

	Inclusion	Exclusion	p-value
	N=9,991	N=4,921	
HLA-A locus matching, no. (%)			
Match	9,365 (94%)	4,290 (87%)	<0.001
Mismatch in the GVH direction	60 (1%)	42 (1%)	
Mismatch in the HVG direction	83 (1%)	51 (1%)	
Bidirectional mismatch	483 (5%)	538 (11%)	
HLA-B locus matching, no. (%)			
Match	9,748 (98%)	4,509 (92%)	<0.001
Mismatch in the GVH direction	12 (0%)	8 (0%)	
Mismatch in the HVG direction	27 (0%)	12 (0%)	
Bidirectional mismatch	204 (2%)	392 (8%)	
HLA-C locus matching, no. (%)			
Match	7,958 (80%)	2,783 (57%)	<0.001
Mismatch in the GVH direction	180 (2%)	108 (2%)	
Mismatch in the HVG direction	168 (2%)	112 (2%)	
Bidirectional mismatch	1,685 (17%)	1,918 (39%)	
HLA-DRB1 locus matching, no. (%)			
Match	7,871 (79%)	2,452 (50%)	<0.001
Mismatch in the GVH direction	121 (1%)	118 (2%)	
Mismatch in the HVG direction	176 (2%)	170 (3%)	
Bidirectional mismatch	1,823 (18%)	2,181 (44%)	
HLA-DQB1 locus matching, no. (%)			
Match	7,842 (78%)		
Mismatch in the GVH direction	143 (1%)		
Mismatch in the HVG direction	155 (2%)		
Bidirectional mismatch	1,851 (19%)		
Cumulative number of allele matching in the GVH direction, no. (%)			
10/10 Match	6,288 (63%)		
9/10 Match	1,529 (15%)		
8/10 or less Match	2,174 (22%)		
Cumulative number of allele matching in the HVG direction, no. (%)			
10/10 Match	6,195 (62%)		
9/10 Match	1,582 (16%)		
8/10 or less Match	2,214 (22%)		
anti HLA antibodies, no. (%)			
Not detected	2,141 (21%)	1,530 (31%)	<0.001
DNSA	453 (5%)	399 (8%)	
DSA	25 (0%)	28 (1%)	
No analysis or missing	7,372 (74%)	2,964 (60%)	
DQB1 typing method, no. (%)			
Missing data for information of HLA and outcomes	0 (0%)	1,151 (23%)	<0.001
Non-existing HLA in Japanese	0 (0%)	275 (6%)	
PCR-SBT	5,210 (52%)	0 (0%)	
MPA	4,781 (48%)	0 (0%)	
Unpredictable	0 (0%)	3,495 (71%)	
Patient age, y	46 (14)	47 (14)	<0.001
Donor age, y	36 (8)	37 (8)	<0.001
Transplant year			
2000-2005	3,011 (30%)	347 (7%)	<0.001
2006-2010	2,820 (28%)	1,278 (26%)	
2011-2018	4,160 (42%)	3,296 (67%)	
Sex match			
Female match	1,564 (16%)	754 (15%)	<0.001
Male match	4,402 (44%)	2,108 (43%)	
Male to female	2,409 (24%)	1,096 (22%)	
Female to male	1,586 (16%)	871 (18%)	
Missing	30 (0%)	92 (2%)	
ABO match			
Match	5,426 (54%)	2,144 (44%)	<0.001
Minor Mismatch	1,939 (19%)	1,144 (23%)	
Major Mismatch	1,632 (16%)	961 (20%)	
Bidirectional Mismatch	954 (10%)	567 (12%)	
Missing	40 (0%)	105 (2%)	
Diagnosis, no. (%)			
AML	3,917 (39%)	1,900 (39%)	<0.001
ALL	1,913 (19%)	869 (18%)	
MDS	1,335 (13%)	749 (15%)	
CML/MPN	920 (9%)	386 (8%)	
ML/MM	1,906 (19%)	1,017 (21%)	
Disease stage, no. (%)			
Standard	5,985 (60%)	2,813 (57%)	<0.001
High	3,730 (37%)	2,015 (41%)	
Missing	276 (3%)	93 (2%)	
Conditioning, no. (%)			
MAC	6,802 (68%)	3,215 (65%)	<0.001
RIC	3,035 (30%)	1,627 (33%)	
Missing	154 (2%)	79 (2%)	
Total body irradiation, no. (%)			
No	2,466 (25%)	1,413 (29%)	<0.001
Yes	7,163 (72%)	3,434 (70%)	
Missing	362 (4%)	74 (2%)	
*in vivo* T cell depletion, no. (%)			
No	9,546 (96%)	4,329 (88%)	<0.001
Yes	445 (4%)	592 (12%)	
GVHD prophylaxis, no. (%)			
MTX	9,423 (94%)	4,544 (92%)	<0.001
MMF	239 (2%)	164 (3%)	
Others	329 (3%)	213 (4%)	
Cell dose, no. (%)			
<2.0	1,964 (20%)	1,038 (21%)	0.380
2.0-3.9	6,421 (64%)	3,194 (65%)	
4.0-	729 (7%)	372 (8%)	
Missing	877 (9%)	317 (6%)	
CMV serostatus, no. (%)			
R-D-	606 (6%)	340 (7%)	0.005
R+D-	2,099 (21%)	1,366 (28%)	
R-D+	699 (7%)	355 (7%)	
R+D+	3,542 (35%)	2,161 (44%)	
Missing	3,045 (30%)	699 (14%)	
HCT-CI, no. (%)			
0	4,166 (42%)	2,497 (51%)	<0.001
1-2	1,678 (17%)	988 (20%)	
3>=	955 (10%)	578 (12%)	
Missing	3,192 (32%)	858 (17%)	
Performance Status, no. (%)			
0-1	8,460 (85%)	4,389 (89%)	<0.001
2-4	757 (8%)	391 (8%)	
Missing	774 (8%)	141 (3%)	

HLA, human leukocyte antigen;, DNSA, donor non-specific antibody; DSA, donor specific antibody; hematopoietic cell transplantation specific comorbidity index; PCR-SBT, polymerase chain reaction-sequencing based typing; MPA, maximum probability algorithm; AML, acute myeloid leukemia; ALL, acute lymphoblastic leukemia; MDS, myelodysplastic syndrome; CML, chronic myeloid leukemia; MPN, myeloproliferative neoplasm; ML, malignant lymphoma; MM, multiple myeloma; MAC, myeloablative conditioning; RIC, reduced intensity conditioning; GVHD, graft-versus-host disease; MTX, methotrexate; MMF, mycophenolate mofetil; CMV, cytomegalovirus; D-, donor seronegative; D+, donor seropositive; R-, recipient seronegative; R+, recipient seropositive; HCT-CI: Hematopoietic cell transplantation-specific comorbidity index.

## Results

### Prediction of HLA-DQB1 Alleles

**Figure 1:**
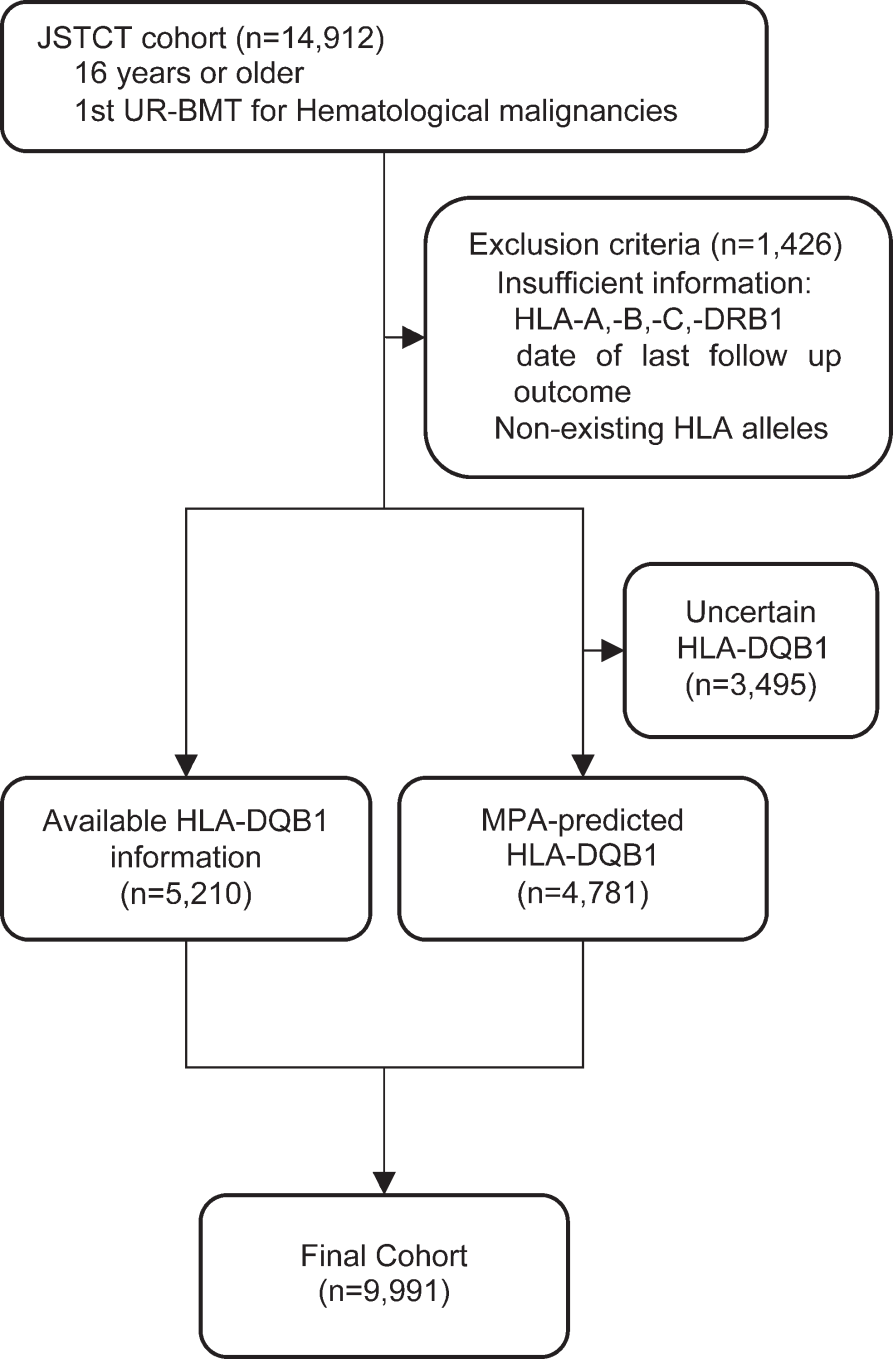
Schematic overview of the inclusion and exclusion of patients.

A schematic workflow for the inclusion and exclusion of our study patients is shown in **Figure 1**. Information on HLA-DQB1 alleles was available for 5,210; 99% of the patients for whom information on HLA-DQB1 alleles of recipient-donor pairs was available were transplanted between 2000 and 2010 because HLA retyping for DQB1 alleles was conducted in the previous study (39). To analyze a recent study cohort, we established MPA for predicting HLA-DQB1, and validated its accuracy by comparison of predicted DQB1 alleles with PCR-SBT-based DQB1 information (**Supplementary Figure S1**). After excluding 1,426 patients with insufficient information for HLA-A, -B, -C, -DRB1, date of last follow up or outcomes and patients for whom patient or donor HLA included non-existing HLA alleles in the Japanese database, of the 5,210 patients with HLA-DQB1 allele information, HLA-DQB1 alleles for 2,916 patients could be predicted by MPA. Importantly, in 2,750 of 2,916 patients (94.3%), MPA-predicted DQB1 alleles were compatible with PCR-SBT-typed DQB1 alleles in both recipient and donor DQB1 alleles (95.0% in matched patients and 92.7% in mismatched patients). In 8,276 patients without PCR-SBT-typed DQB1 alleles, 3,640 out of 4,542 (80.1%) 8/8 HLA-matched recipient-donor pairs and 1,141 out of 3,734 (30.6%) HLA-mismatched recipient-donor pairs could be predicted using MPA. Finally, we enrolled 9,991 patients in this study.

### Patient Characteristics

The median follow-up period for survivors was 6.3 years (interquartile range [IQR], 2.5–9.2 years) after HSCT (**Table 1**). The most common indication for HSCT was acute myeloid leukemia (AML; n=3,917, 39.2%) followed by acute lymphoblastic leukemia (ALL; n=1,913, 19.2%), mature lymphoid malignancies (malignant lymphoma or multiple myeloma (ML/MM); n=1,906, 19.1%), myelodysplastic syndrome (MDS; n=1,335, 13.4%), and chronic myeloid leukemia or other myeloproliferative neoplasms (CML/MPN; n=920, 9.2%).

Overall, 6,288, 1,529, and 2,174 patients were transplanted from 10/10, 9/10, and 8/10 or less than 8/10 matched unrelated donors In the GVH direction, and 6,195, 1,582, and 2,214 patients were transplanted from 10/10, 9/10, and 8/10 or less than 8/10 matched unrelated donors In the HVG direction, respectively. The number of allele-mismatched recipient-donor pairs was 626 for the HLA-A locus, 243 for the HLA-B locus, 2,033 for the HLA-C locus, and 2,120 for the HLA-DRB1 locus. In addition, 1,351 patients with PCR-SBT-typed HLA-DQB1 information and 500 patients with MPA-predicted HLA-DQB1 alleles were transplanted from HLA-DQB1 mismatched donors. In total, 2,149 recipient-donor pairs had allele mismatches at the HLA-DQB1 locus. Percentages of allele matched patients were higher in final cohort than excluded patients at the HLA-A, -B, -C, and -DRB1 locus (final cohort *vs* excluded patients: 94% *vs* 87%, 98% *vs* 92%, 80% *vs* 57%, 79% *vs* 50%, respectively).

Information for donor specific antibodies was available for 2,619 patients and 25 patients (0.25%) had donor specific antibodies. 445 patients (4.5%) received T cell depletion using anti-thymocyte globulin.

### Distributions of HLA Epitope Mismatching

**Figure 2:**
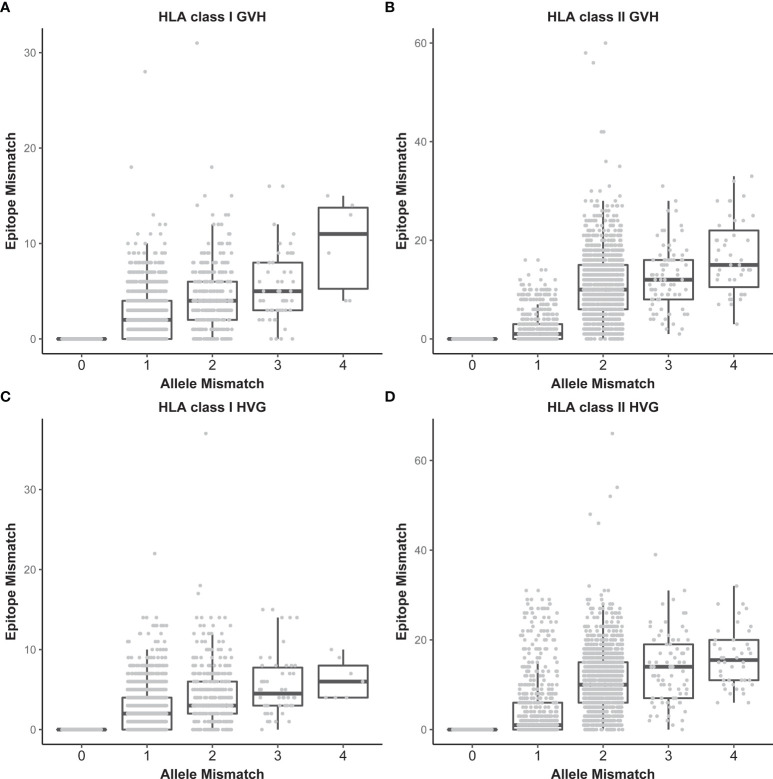
Relationships between the number of HLA allele mismatches and that of HLA epitope mismatches. HLA class I in the GVH direction **(A)**, HLA class II in the GVH direction **(B)**, HLA class I in the HVG direction **(C)**, and HLA class II in the HVG direction **(D)**. Each dot represents an individual recipient-donor pair.

The association of EMM with HLA allele mismatching is shown in **Figure 2**. The median number of HLA class I EMM was 0 in both the GVH and HVG directions (range, 0-30, 0-37, respectively). The median number of HLA class II EMM was 1 in both the GVH and HVG directions (range, 0-60, 0-54, respectively). We divided patients into low EMM and high EMM groups using the median value for each epitope matching as a threshold: 1,697 patients were in the HLA class I high EMM group (number of class I EMM >=1) and 1,897 patients were in the HLA class II high EMM group (number of class II EMM >=2).

### Impact of HLA Allele, Antigen and Epitope Mismatching on HSCT Outcomes

**Figure 3:**
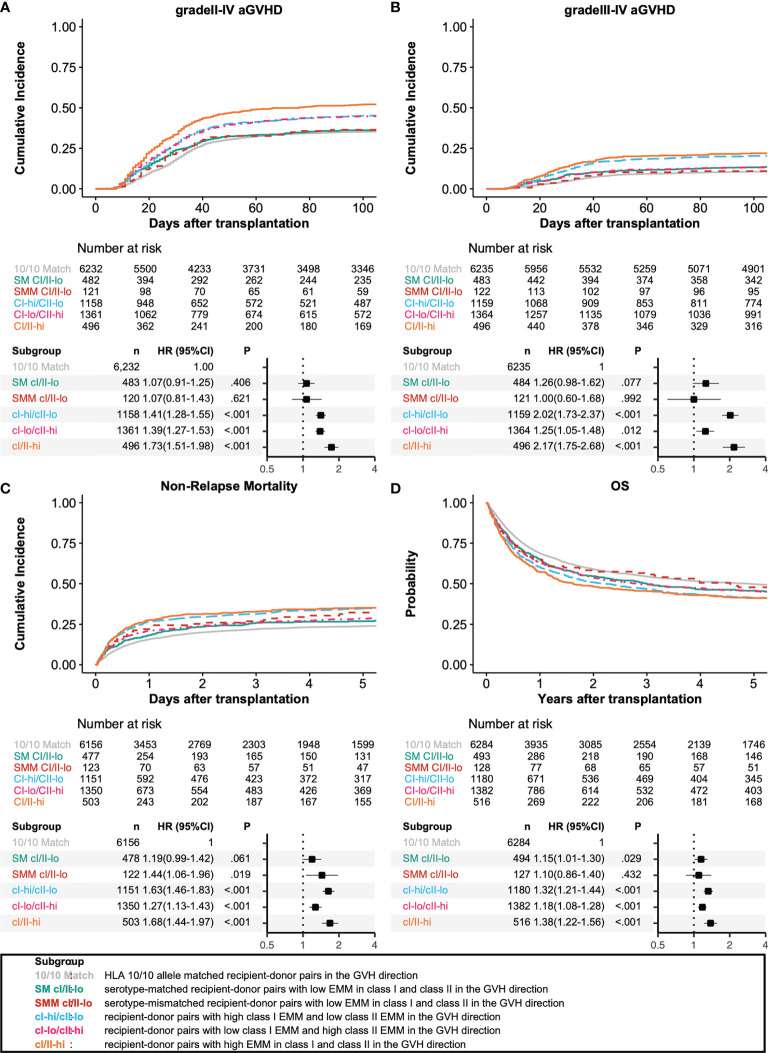
Impact of antibody-identified HLA epitopes on HSCT outcomes in the GVH direction. Adjusted survival and cumulative incidence curves (upper) and forest plot (lower) are shown for grade II-IV acute GVHD **(A)**, grade III-IV acute GVHD **(B)**, non-relapse mortality **(C)**, and overall survival **(D)**. SM CI/II-lo, SMM CI/II-lo, CI-hi/CII-lo, CI-lo/CII-hi, and CI/II-hi represent serotype-matched recipient-donor pairs with low EMM in class I and class II, serotype-mismatched recipient-donor pairs with low EMM in class I and class II, recipient-donor pairs with high class I EMM and low class II EMM, recipient-donor pairs with low HLA EMM in class I and low EMM in class II, and recipient-donor pairs with high EMM in class I and class II, respectively.

**Figure 4:**
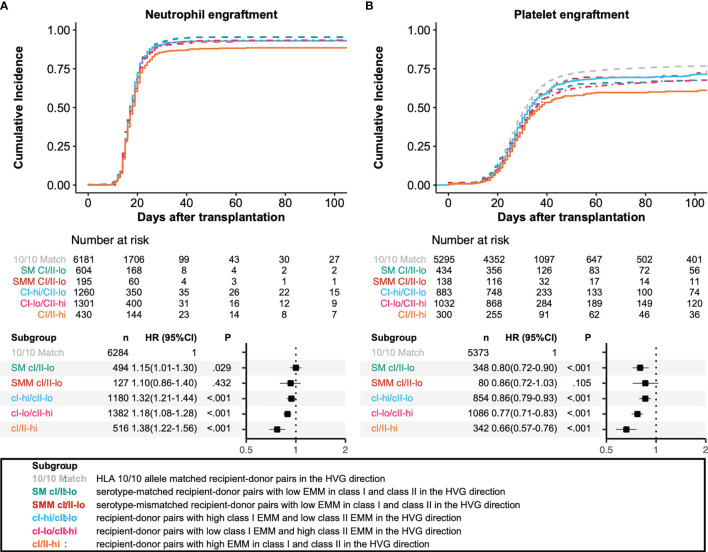
Impact of antibody-identified HLA epitopes on HSCT outcomes in the HVG direction. Adjusted survival and cumulative incidence curves (upper) and forest plot (lower) are shown for neutrophil engraftment **(A)** and platelet engraftment **(B)**. SM CI/II-lo, SMM CI/II-lo, CI-hi/CII-lo, CI-lo/CII-hi, and CI/II-hi represent serotype-matched recipient-donor pairs with low EMM in class I and class II, serotype-mismatched recipient-donor pairs with low EMM in class I and class II, recipient-donor pairs with high class I EMM and low class II EMM, recipient-donor pairs with low HLA EMM in class I and low EMM in class II, and recipient-donor pairs with high EMM in class I and class II, respectively.

We analyzed the associations among HLA allele mismatching, antigen/serotype mismatching, and EMM with transplantation outcomes in a multivariate analysis (**Figures 3**, **4**). In the GVH direction, compared with an HLA 10/10 allele matched group as a reference group, the CI-hi/CII-lo group and CI/II-hi group showed significantly higher incidences of grade III-IV aGVHD (HR 2.02, 95% CI 1.73-2.37; HR 2.17, 95% CI 1.75-2.68, respectively). Three higher EMM groups, the CI-hi/CII-lo group, CI-lo/CII-hi group and CI/II-hi group, showed significantly higher risk of grade II-IV aGVHD, NRM and lower OS (grade II-IV aGVHD: HR 1.41, 95% CI 1.28-1.55; HR 1.39, 95% CI 1.27-1.53; HR 1.73, 95% CI 1.51-1.98, respectively; NRM: HR 1.63, 95% CI 1.46-1.83; HR 1.27, 95% CI 1.13-1.43; HR 1.68, 95% CI 1.44-1.97, respectively; OS: HR 1.32, 95% CI 1.21-1.44; HR 1.18, 95% CI 1.08-1.28; HR 1.38, 95% CI 1.22-1.56, respectively). Only the CI-hi/CII-lo group showed a statistically significant association with a reduced incidence of relapse, and none of the five subgroups of recipient-donor pairs with AMM showed a statistically significant association with a higher risk for cGVHD and excGVHD. In the HVG direction, the CI-lo/CII-hi group and CI/II-hi group showed a significantly longer time to neutrophil engraftment than the reference group (HR 0.89, 95%CI 0.84-0.94; HR 0.77, 95%CI 0.69-0.86, respectively). A longer time to platelet engraftment was observed in the SM CI/II-lo group and three eplet-mismatched subgroups (HR 0.80, 95%CI 0.72-0.90; HR 0.86, 95%CI 0.79-0.93; HR 0.77, 95%CI 0.71-0.83; HR 0.66, 95%CI 0.57-0.76, respectively).

The effect of all the other covariates than HLA eplet matching on transplantation outcomes is shown in **Supplementary Table S2**. Older donor age, male patients or sex mismatching, and higher disease stage showed a higher risk for grade III-IV aGVHD and *in vivo* T cell depletion showed significantly lower incidence of grade III-IV aGVHD.

A subgroup analysis limited to HLA 10/10 or 9/10 matched recipient-donor pairs was subsequently carried out (**Table 2**). Compared with 10/10 matched recipient-donor pairs, class I high EMM in the GVH direction was significantly associated with higher risk of grade III-IV aGVHD, NRM and lower OS (HR 1.83, 95%CI 1.51-2.20; HR 1.63, 95%CI 1.43-1.86; HR 1.30, 95%CI 1.18-1.44, respectively).

**Table 2:** Multivariate analysis for gradeIII-IV aGVHD, Non-Relapse Mortality and Overall Survival in 10/10 or 9/10 HLA matched recipient-donor pairs.

Category*	gradeIII-IV aGVHD				Non-Relapse Mortality				Overall Survival			
	n	HR	95%CI	P	n	HR	95%CI	P	n	HR	95%CI	P
HLA allele matching												
10/10	6,235	1.00			6,156	1.00			6,284	1.00		
9/10												
SM CI/II-lo	388	1.31	0.99-1.74	.058	380	1.22	1.00-1.49	.046	394	1.14	0.99-1.31	.069
SMM CI/II-lo†	95	0.99	0.54-1.82	.982	96	1.32	0.91-1.90	.139	101	1.09	0.83-1.43	.555
CI-hi/CII-lo†	835	1.81	1.50-2.18	<.001	826	1.63	1.42-1.86	<.001	845	1.30	1.18-1.44	<.001
CI-lo/CII-hi†	183	0.99	0.63-1.55	.966	183	1.24	0.93-1.64	.144	186	1.11	0.91-1.36	.293

*GVH direction.

†Irrespective of serotype matching status.

aGVHD, acute graft-versus-host disease; SM CI/II-lo, serotype-matched recipient-donor pairs with low EMM in class I and class II; SMM CI/II-lo, serotype-mismatched recipient-donor pairs with low EMM in class I and class II; CI-hi/CII-lo, recipient-donor pairs with high class I EMM and low class II EMM; CI-lo/CII-hi, recipient-donor pairs with low class I EMM and high class II EMM.

### Identifying High-Risk Donor-Recipient Pairs Based on Eplet Mismatching at the HLA-C Locus

Next, we investigated the association of HLA class I epitopes derived from an individual locus with severe aGVHD and NRM. We also checked correlation of allele and eplet matching of each locus and found that allele and eplet matching status for each of HLA class I locus is poorly correlated with that for other HLA class I locus and for HLA class II locus (**Supplementary Table S3**). Compared with HLA allele-matched recipient-donor pairs, HLA-A EMM and HLA-C EMM were associated with higher risks for severe aGVHD and NRM (**Supplementary Table S4**). HLA-DRB1 EMM was also associated with higher risk for grade II-IV aGVHD but not for grade III-IV aGVHD and NRM. Recipient-donor pairs with HLA-C EMM accounted for 94.5% (n=1,603) of those with HLA class I EMM. We further investigated the impact of HLA-C EMM on severe aGVHD in relation to other known high-risk mismatch patterns. All killer immunoglobulin-like receptor (KIR)-ligand mismatched recipient-donor pairs (n=376) had HLA-C EMM. In a multivariate analysis, patients with KIR-ligand mismatches and EMM did not show a higher incidence of grade III-IV aGVHD compared with KIR-ligand-matched patients with EMM (HR 0.96, 95% CI 0.74–1.25).

**Figure 5:**
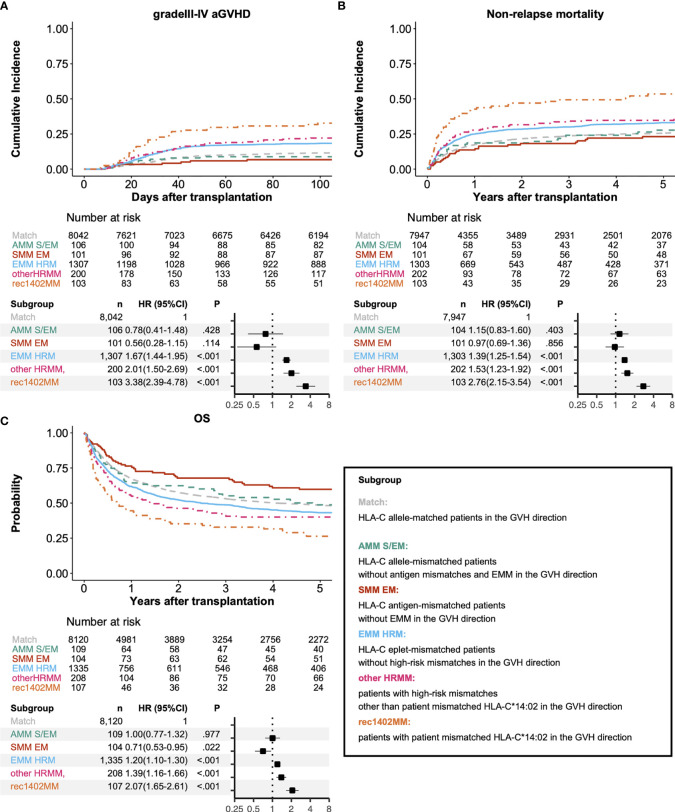
Association between clinical outcomes and matching of alleles, serotypes, epitopes and high-risk mismatches at the HLA-C locus. Adjusted survival and cumulative incidence curves (upper) and forest plot (lower) for relative risks of grade III-IV acute GVHD **(A)**, non-relapse mortality **(B)**, and overall survival **(C)**. Match, AMM S/EM, SMM EM, EMM HRM, other HRMM, and rec1402MM represent HLA-C allele-matched patients, HLA-C allele-mismatched patients without antigen mismatches and EMM, HLA-C antigen-mismatched patients without EMM, HLA-C eplet-mismatched patients without high-risk mismatches, Patients with high-risk mismatches other than patient mismatched HLA-C*14:02, and Patients with patient mismatched HLA-C*14:02, respectively.

In addition to the known high-risk mismatch patterns in the Japanese cohort, EMM was associated with a higher risk for grade III-IV aGVHD (**Figure 5**, compared with HLA-C allele-matched patients (Match), HLA-C allele-mismatched patients without antigen mismatches, and EMM (AMM S/EM): HR 0.78, 95% CI 0.41–1.48; HLA-C antigen-mismatched patients without EMM (SMM EM): HR 0.56, 95% CI 0.28–1.15; HLA-C eplet-mismatched patients without high-risk mismatches (EMM HRM): HR 1.67, 95% CI 1.44–1.95; other HRMM: HR 2.01, 95% CI 1.50–2.69; rec1402MM: HR 3.38, 95% CI 2.39–4.78). HLA-C eplet-mismatched patients without high-risk mismatches also showed higher risk of NRM and lower OS than HLA-C allele-matched patients (NRM: HR 1.39, 95% CI 1.25–1.54; OS: HR 1.20, 95% CI 1.10–1.30). Subgroup analysis showed a higher incidence of grade III-IV aGVHD in the EMM HRM group compared with the reference group regardless of the year of transplant (**Supplementary Figure S2**, patients receiving transplantation from 2000 to 2010: HR 1.62, 95% CI 1.37–1.92; patients receiving transplantation from 2011 to 2018: HR 2.02, 95% CI 1.46–2.79).

## Discussion

This study sought to evaluate the association of HLA EMM with outcomes after UR-BMT for Japanese patients with hematologic malignancies. Previous studies found amino acid substitutions that were associated with a high risk for a poor outcome after HSCT in a Japanese cohort (13, 17). These studies mainly focused on the direct recognition of mismatched amino-acid sequences by HLA molecules expressed by T cells. We found a significant association of antibody-identified HLA epitopes quantified by HLAMM with HSCT outcomes and a high-risk HLA-C mismatch pattern other than amino-acid substitutions that are known to be associated with a high risk for severe aGVHD, leading to a higher incidence of NRM and poor OS after transplantation.

We found that class I EMM in the GVH direction had a negative impact on the incidence of severe aGVHD and class II EMM in the HVG direction had a negative impact on neutrophil engraftment. This study further elucidated the significant effect of EMM in the GVH direction at the HLA-C locus on severe aGVHD leading to lower OS for the first time. We should consider several points to understand the discrepancies between the results in this study and previous findings.

First, the donor source and GVHD prophylaxis might affect immune reconstitution and alloimmunity after transplantation. We found that half and one thirds of HLA mismatched patients had epitope mismatching in HLA class I and class II, respectively, and these frequencies are much lower than those in haploidentical transplantation (29). PTCy can effectively prevent GVHD, so it has been widely used and well-established GVHD prophylaxis for haplo-HSCT. However, the efficacy of PTCy for UR-BMT is not determined and most of the patients in our cohort did not receive PTCy Although preventive effect of PTCy is considered to be owing to suppression of effector T cells, cyclophosphamide can also prevent B cell immunity and might affect antibody-mediated alloimmunity after transplantation (40). The sample size is also essential for interpreting the wide variety of disparities in HLA alleles and haplotypes, so we selected a retrospective study design. Both Duquesnoy et al. and our study showed a tendency for a higher risk of aGVHD in HLA class I mismatched recipient-donor pairs, and the larger cohort in our study might have contributed to this finding being statistically significant (24). In addition to the differences in donor source, GVHD prophylaxis and sample size, demographic distributions of HLA alleles and haplotypes might influence the heterogeneous effect of HLA EMM. Haplostats based on the NMDP database was used for HLA-DQB1 prediction in the previous study of haploidentical HSCT (29). However, the frequency distributions of HLA alleles and haplotypes in Japanese populations were different from those in Asian-Pacific populations in the NMDP database (41, 42). Moreover, a comparison of CIBMTR and TRUMP data showed lower incidences of grade III-IV acute GVHD and relapse in a Japanese population than in American Caucasians (43). Further study is warranted to investigate the relationship between eplet mismatching and HSCT outcomes under various ethnic backgrounds and clinical practices.

Our findings demonstrated the feasibility of HLAMM-based mismatched antibody epitope detection in HSCT. At the HLA-C locus, all KIR-ligand mismatched recipient-donor pairs also had HLA-C EMM. Morishima et al. reported that patient mismatched HLA-C*14:02 is a critical factor in severe acute GVHD regardless of KIR-ligand mismatching (17). Our findings might suggest that the higher incidences in KIR-ligand mismatching in UR-BMT is partially explained by antibody-mediated alloimmunity in patients without high-risk HLA-C mismatching. Delbos et al. reported that HLA class II donor-derived antibody was associated with higher risks for acute and chronic GVHD (44). Several reports have demonstrated that B cell depletion by Rituximab reduced the incidence of aGVHD (45, 46). Although HLA eplet-composing amino acid mismatching might be identified as epitopes not only by antibody but also by HLA, which can cause T cell-mediated alloimmunity, our findings suggest that B cell immunity plays a role in the pathogenesis of aGVHD. T cell-directed immune suppression is often used for patients transplanted from mismatched related or unrelated donors, but we should reconsider the efficacy of B cell-directed immune suppression on HSCT outcomes.

This study has some limitations associated with our study design. We predicted haplotypes and DQB1 alleles from haplotype frequency data from a family study in a Japanese population using a maximum probability algorithm. The predictive accuracy of this algorithm was validated by a compatibility of 94.3% compared with PCR-SBT-based detection of DQB1. However, minor DQB1 alleles or haplotype pairs were ignored. Because MPA excluded more than two-thirds of the patients transplanted from HLA-mismatched donors, we accounted for type of HLA-DQB1 alleles (PCR-SBT-typed or MPA-predicted) as a covariate in the multivariate analysis. However, a selection bias due to MPA might still influence the interpretation of this study. For example, we did not find a significant association of HLA-B AMM with severe aGVHD or NRM. Previous studies have reported that HLA-B antigen and allele mismatches significantly affect outcomes after UR-BMT in a Japanese cohort, which might cause a preference for HLA-C or -DRB1 locus mismatching over HLA-A or -B locus mismatching (3). The discrepancies between this study and previous studies might be due to the relatively small percentage of HLA-B mismatched patients in our cohorts. HLA-DRB1 mismatching also showed significant association with grade II-IV aGVHD although HLA-DRB1 mismatching did not show significant association with grade III-IV aGVHD and NRM. HLA-DRB1 mismatching was highly correlated with HLA-DQB1 mismatching, so the association of HLA-DRB1 mismatching with HSCT outcomes might be underestimated. Moreover, we could not investigate the effect of HLA-DPB1 mismatching because haplotype-based prediction could not be applied to HLA-DPB1 locus in which T cell epitope matching is associated with aGVHD (20, 47–49). Thus, further study is needed to understand the effects of HLA-A, HLA-B, HLA-DRB1 and HLA-DPB1 locus disparities on HSCT outcomes. 74% of patients lacked information about donor-specific antibody before transplantation and no patients had information about donor-derived antibody. To validate the effect of antibody-mediated alloimmunity on transplantation outcomes, we should understand the significance of donor-specific and donor-derived antibody before and after transplantation.

In conclusion, we found that class I EMM in the GVH direction negatively affected the incidence of aGVHD, leading to transplantation-related mortality. At the HLA-C locus, epitope-mismatched recipient-donor pairs are non-permissive mismatched patterns along with known high-risk amino acid substitutions. Our findings might be helpful for clinicians in selecting permissive donors from alternative donor options.

## Data Availability Statement

The data analyzed in this study is subject to the following licenses/restrictions: The data that support the findings of this study are available from the corresponding author upon reasonable request. Requests to access these datasets should be directed to JK, jkanda16@kuhp.kyoto-u.ac.jp.

## Ethics Statement

The studies involving human participants were reviewed and approved by Kyoto University Hospital, the Data Management Committees of the Japanese Society for Transplantation and Cellular Therapy (JSTCT) and the Japanese Data Center for Hematopoietic Cell Transplantation (JDCHCT). Written informed consent to participate in this study was provided by the participants’ legal guardian/next of kin.

## Author Contributions

MI and JK planned the study. MI, JK, and HT performed HLA-DQB1 prediction. TSa, TSh, ND, TF, YO, TE, NU, YuK, KK, TA, SO, MO, YoK, TI, YA, and SM organized and collected the clinical data and samples for transplantation. MI and JK conducted the statistical analysis. MI and JK wrote the paper. All authors contributed to the article and approved the submitted version.

## Funding

This work was supported in part by AMED under Grant Number JP18pc0101031, JSPS KAKENHI Grant Number 18K08325 and 21K08391 (JK), and the Takeda Science Foundation (JK).

## Conflict of Interest

JK reports grants from AMED (Grant Number: JP18pc0101031), grants from JSPS KAKENHI (Grant Number 18K08325 and 21K08391), grants from Takeda Science Foundation, during the conduct of the study.

The remaining authors declare that the research was conducted in the absence of any commercial or financial relationships that could be construed as a potential conflict of interest

## Publisher’s Note

All claims expressed in this article are solely those of the authors and do not necessarily represent those of their affiliated organizations, or those of the publisher, the editors and the reviewers. Any product that may be evaluated in this article, or claim that may be made by its manufacturer, is not guaranteed or endorsed by the publisher.
