# Supplementary material for: Impact of HLA Epitope Matching on Outcomes After Unrelated Bone Marrow Transplantation
Source: Front Immunol. 2022 Mar 3;13:811733. doi: 10.3389/fimmu.2022.811733 (PMC8928463; doi:10.3389/fimmu.2022.811733)

Supplemental Figure S1.
Example of estimation of HLA-DQB1 for one patient based on maximum probability algorithms (MPA)

example HLA typing: A*26:03,*31:01; C*03:03, *03:04; B*15:01, *40:02; DRB1*04:05,*15:01

HLA haplotype estimation from HLA-A, -B, -C, -DR haplotype frequency

HLA-A, -C, -B, -DRB1 Haplotype (A)	Haplotype frequency* (A)	HLA-A, -C, -B, -DRB1 Haplotype (B)	Haplotype frequency* (B)	Frequency† (AxB)	Likelihood Ratio‡
***26:03-*03:03-*15:01-*15:01**	**0.344%**	***31:01-*03:04-*40:02-*04:05**	**0.120%**	**0.00041280%**	**90.8%**
*26:03-*03:03-*15:01-*04:05	0.052%	*31:01-*03:04-*40:02-*15:01	0.047%	0.00002444%	5.4%
*26:03-*03:04-*40:02-*04:05	0.010%	*31:01-*03:03-*15:01-*15:01	0.136%	0.00001360%	3.0%
*26:03-*03:04-*40:02-*15:01	0.005%	*31:01-*03:03-*15:01-*04:05	0.047%	0.00000235%	0.5%
*26:03-*03:04-*15:01-*04:05	0.005%	*31:01-*03:03-*40:02-*15:01	0.010%	0.00000050%	0.1%
*26:03-*03:03-*40:02-*04:05	0.005%	*31:01-*03:04-*15:01-*15:01	0.005%	0.00000025%	0.1%
*26:03-*03:04-*15:01-*15:01	0.005%	*31:01-*03:03-*40:02-*04:05	0.005%	0.00000025%	0.1%
*26:03-*03:03-*40:02-*15:01	0.005%	*31:01-*03:04-*15:01-*04:05	0.005%	0.00000025%	0.1%

Estimation for HLA-DQB1 alleles from HLA-B, -DR, -DQ haplotype frequency

HLA-B, -DRB1, -DQB1 Haplotype (A)	DQB1 allele (A)	Haplotype frequency* (A)	Likelihood ratio §
*15:01-*15:01-*06:02	*06:02	0.568%	100.0%

HLA--B, -DRB1, -DQB1 Haplotype (B)	DQB1 allele (B)	Haplotype frequency* (B)	Likelihood ratio §
*40:02-*04:05-*04:01	*04:01	0.435%	81.3%
*40:02-*04:05-*03:02	*03:02	0.067%	12.5%
*40:02-*04:05-*05:02	*05:02	0.033%	6.2%

*Haplotype frequency was extracted from a family study in a Japanese population (Ikeda N, et al. Tissue Antigens 2015; 85: 252-259.). The frequency of private HLA haplotype was set as 0.005%, which was the lower limit that can be calculated from the dataset of the Japanese population.

†Frequency was calculated by multiplying haplotype frequency of A and B.

‡Likelihood ratio was calculated by the frequency of each pattern divided by the sum of all frequencies of possible patterns for HLA-A, -C, -B, -DRB1 haplotype A and B.

§Likelihood ratio was calculated by the frequency of each pattern divided by the sum of all frequencies of possible patterns for HLA-B, -DRB1, -DQB1 haplotype A or B.

Supplementary Figure S2. Adjusted survival and cumulative incidence curves (left) and forest plot (right) for relative risk of grade III-IV acute GVHD according to matching of HLA-C alleles, serotypes, epitopes and high-risk mismatches in patients receiving transplantation from 2000 to 2010 (A) and from 2011 to 2018 (B) are shown. Match, AMM S/EM, SMM EM, EMM HRM, other HRMM, rec1402MM represent HLA-C allele-matched patients, HLA-C allele- mismatched patients without antigen mismatches and EMM, HLA-C antigen-mismatched patients without EMM, HLA-C eplet-mismatched patients without high-risk mismatches, Patients with high-risk mismatches other than patient mismatched HLA-C*14:02, and Patients with patient mismatched HLA-C*14:02, respectively.

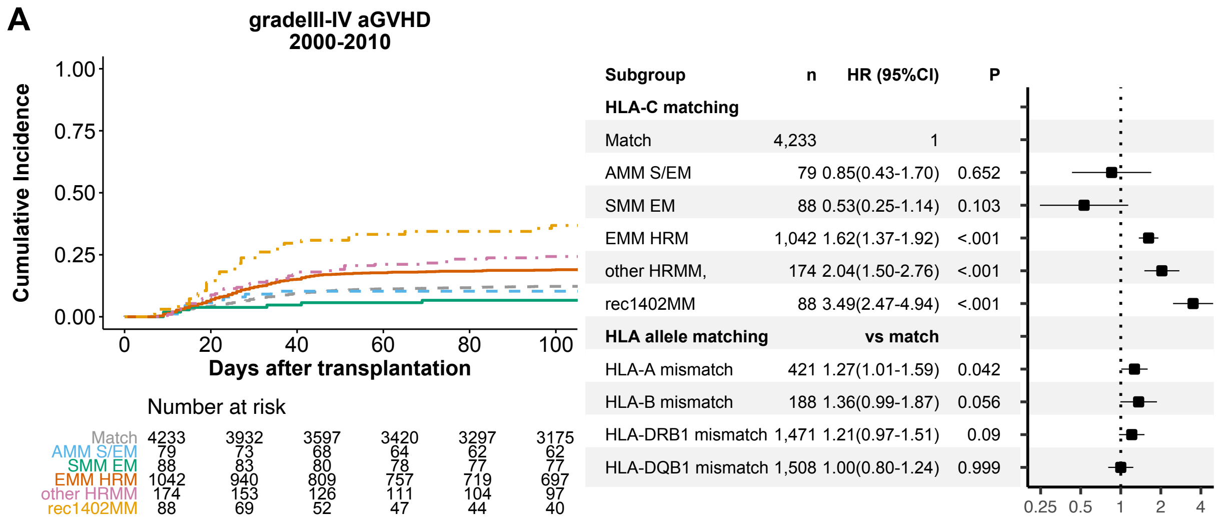

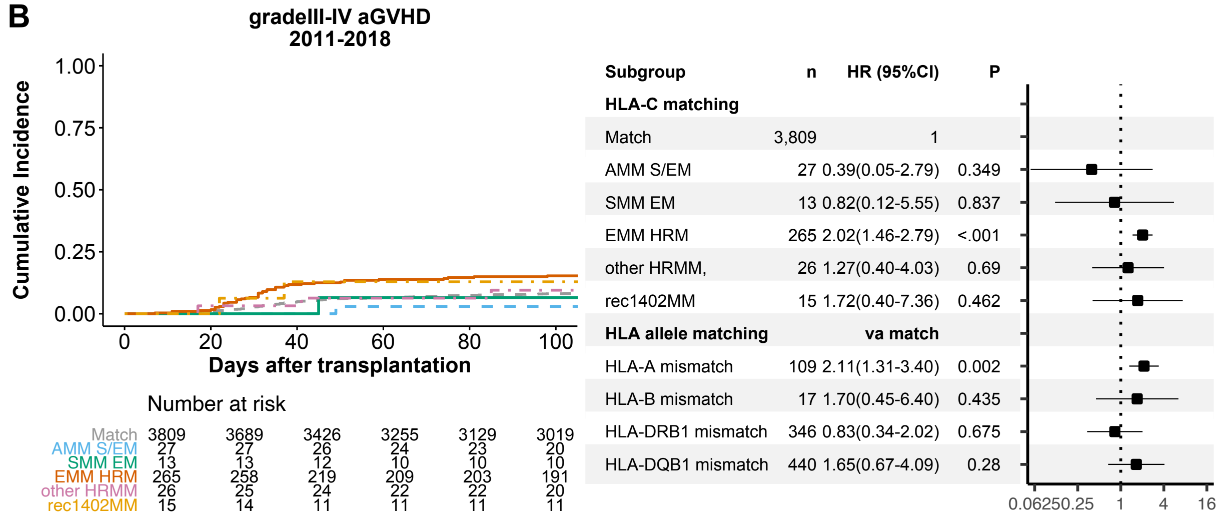

Supplement: Supplementary file 1 [file Image_1.pdf]
